# Oleanolic Acid’s Semisynthetic Derivatives HIMOXOL and Br-HIMOLID Show Proautophagic Potential and Inhibit Migration of HER2-Positive Breast Cancer Cells In Vitro

**DOI:** 10.3390/ijms222011273

**Published:** 2021-10-19

**Authors:** Natalia Magdalena Lisiak, Izabela Lewicka, Mariusz Kaczmarek, Jacek Kujawski, Barbara Bednarczyk-Cwynar, Lucjusz Zaprutko, Blazej Rubis

**Affiliations:** 1 Department of Clinical Chemistry and Molecular Diagnostics, Poznan University of Medical Sciences, 49 Przybyszewskiego Str., 60-355 Poznan, Poland; izabela.lewickaa@gmail.com (I.L.); blazejr@ump.edu.pl (B.R.); 2 Department of Cancer Immunology, Chair of Medical Biotechnology, Poznan University of Medical Sciences, Garbary 15 Str., 61-866 Poznan, Poland; markacz@ump.edu.pl; 3 Gene Therapy Laboratory, Department of Cancer Diagnostics and Immunology, Greater Poland Cancer Centre, Garbary 15 Str., 61-866 Poznan, Poland; 4 Department of Organic Chemistry, Poznan University of Medical Sciences, 6 Grunwaldzka Str., 60-780 Poznan, Poland; jacekkuj@ump.edu.pl (J.K.); bcwynar@ump.edu.pl (B.B.-C.); zaprutko@ump.edu.pl (L.Z.)

**Keywords:** breast cancer, HER2, oleanolic acid, oleanolic acid derivatives, HIMOXOL, Br-HIMOLID, autophagy, migration

## Abstract

Approximately 20–30% of the diagnosed breast cancers overexpress the human epidermal growth factor receptor 2 (HER2). This type of cancer is associated with a more aggressive phenotype; thus, there is a need for the discovery of new compounds that would improve the survival in HER2-positive breast cancer patients. It seems that one of the most promising therapeutic cancer strategies could be based on the biological activity of pentacyclic triterpenes’ derivatives and the best-known representative of this group, oleanolic acid (OA). The biological activity of oleanolic acid and its two semisynthetic derivatives, methyl 3-hydroxyimino-11-oxoolean-12-en-28-oate (HIMOXOL) and 12α-bromo-3-hydroxyimonoolean-28→13-olide (Br-HIMOLID), was assessed in SK-BR-3 breast cancer cells (HER2-positive). Viability tests, cell cycle assessment, evaluation of apoptosis, autophagy, and adhesion/migration processes were performed using MTT, clonogenic, cytofluorometry, Western blot, and qPCR. Both derivatives revealed higher cytotoxicity in studied breast cancer cells than the maternal compound, OA. They also decreased cell viability, induced autophagy, and (when applied in sub-cytotoxic concentrations) decreased the migration of SK-BR-3 cells.This study is the first to report the cytostatic, proautophagic (mTOR/LC3/SQSTM/BECN1 pathway), and anti-migratory (integrin β1/FAK/paxillin pathway) activities of HIMOXOL and Br-HIMOLID in HER2-positive breast cancer cells.

## 1. Introduction

Breast cancer is one of the most common neoplasms among women with constantly growing statistics of morbidity and mortality that indicate a lack of effective diagnostic and prognostic tools [1]. Similarly, one of the most important challenges in the area of treatment is the development of personalized therapy suitable for each type of cancer. Importantly, approximately 20–30% of the diagnosed breast cancers show overexpression of human epidermal growth factor receptor 2 (HER2) [2]. The signaling cascades activated in HER2 overexpressing breast cancer are involved in a diverse range of cellular processes such as cell growth, proliferation, survival, differentiation, angiogenesis, and invasion [3]. HER2 is a negative prognostic factor in invasive breast cancer, responsible for extensive metastatic progression [4]. In-depth analysis of the molecular mechanisms underlying HER2-positive cancer development has led to the identification of series of HER2-targeting agents. Consequently, the standard of care for HER2-positive breast cancer was amended [5]. However, all the current anticancer strategies face similar obstacles, i.e., resistance to drugs, low efficacy, and negative adverse effects. Thus, there is a need for strengthening the effect of cancer drugs by using compounds that would constitute adjuvant therapy support. For a long time already, we have been trying to identify natural-derived compounds that would contribute to the higher efficacy of cancer therapy and diminish adverse effects. Long years of research on the biological activity of OA and its derivatives indicated their anticancer potential [6]. This effect has been observed in different cancer types including breast cancer cells, both in vitro and in vivo. Importantly, OA not only revealed antitumor effects but also showed low toxicity in normal cells that make it a very promising anticancer agent [7]. We investigated the possibility to obtain OA derivatives that would be more efficient in cancer targeting. Importantly, we already showed that two of them, methyl 3-hydroxyimino-11-oxoolean-12-en-28-oate (HIMOXOL) and 12α-bromo-3-hydroxyimonoolean-28→13-olide (Br-HIMOLID) revealed cytotoxic activity against ER/PR-positive and HER2-negative (MCF7 and T-47D), and ER/PR/HER2-negative breast cancer cells (MDA-MB-468, MDA-MB-231) [8,9]. These compounds are semisynthetic analogs of OA, modified at positions C3, C11, and C28, and not much is known about their biological activity in HER2-positive breast cancer cells. Results presented in other studies in HER2-overexpressing cell lines revealed that other triterpenoids, naturally occurring (betulinic acid), but also synthetic OA’s derivative (2-cyano-3, 12-dioxooleana-1,9(11)-dien-28-oic acid, CDDO) suppress both tumor cell growth and colony formation and also activate apoptosis [10,11]. As suggested in our previous studies, HIMOXOL and Br-HIMOLID might contribute to the induction of cancer cell death, especially to apoptosis, but also to autophagy, which constitutes one of the key cellular homeostasis mechanisms [12]. Autophagy as a “double-edged sword” is engaged in cancer cell elimination, but also tumor survival, growth, and cancer progression. Thus, modulation of this process might become a promising strategy in combating cancer [13]. Importantly, our previous studies revealed the anti-migratory and anti-invasive potential of studied compounds in MCF7 and MDA-MB-231 cells [8,14]. Since both, tumor angiogenesis and metastasis, depend on cell migration, compounds that show anti-migratory potential seem to be of special interest [15]. Furthermore, some OA derivatives are suspected to play a role of HER2-positive cancer cells modulators, which is associated with their capability to repress the activation of downstream signaling molecules, like mTOR, exhibiting the therapeutic potential of these compounds [16,17]. Consequently, we performed some detailed in silico studies that indicated OA and its derivatives capable to act via HER2 mediated signaling. Thus, HER2 positive breast cancer cells SK-BR-3 were chosen for the verification of the biological potential of indicated compounds to modulate breast cancer cells’ survival, invasive, and metastatic potential.

## 2. Results

### 2.1. Computational Analysis

Molecular docking was used to elucidate whether the influence of HIMOXOL, Br-HIMOLID, or OA acid on HER2 activation is due to a specific interaction with the kinase domain of Human HER2 (erbB2) protein. For this purpose, the crystal structure of the kinase domain of human HER2 (erbB2) (PDB entry: 3PP0) was selected based on the literature data [18,19].

**Figure 1:**
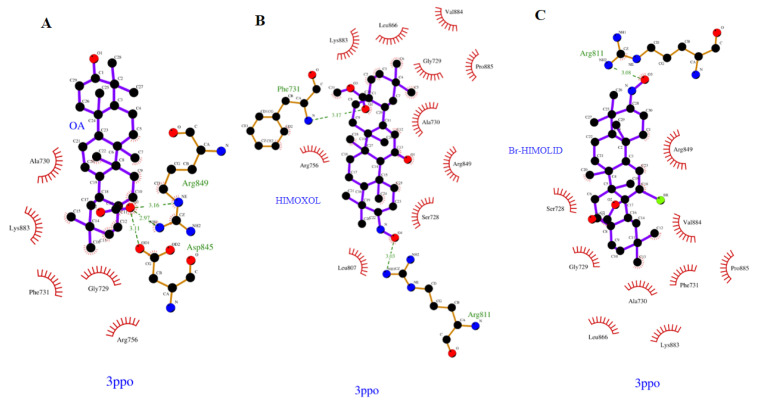
The first poses of the docked OA (**A**), HIMOXOL (**B**), and Br-HIMOLID (**C**) ligands with surroundings amino acids within the cavity (LigPlot+ v.2.2 software [22,23]).

The mutual ligand–amino acid interactions are crucial for the biological effect. We assumed that the studied derivatives might affect particular amino acids that consequently lead to the formation of different orientations within the protein cavity during the docking protocol. Nine poses were obtained for each of the analyzed, previously optimized (Gaussian 16 C.01 program [20] ligands during the docking procedure (AutoDock Vina package [21]) from which the first poses had the most negative value of binding affinity as follows (Figure 1): −6.700, −6.400 and −6.800 kcal mol^−1^ for OA (A), HIMOXOL (B), and Br-HIMOLID (C), respectively. Analyzing the docked derivatives to the 3PPO.pdb protein, we noticed that from the standpoint of molecular mechanics (MM) the most favorable ligand appeared to be oleanane derivative containing the bromine atom (**C**) within its structure.

Based on the literature data, the most interacting amino acid residues surrounding this cavity were selected, i.e., Phe731, Arg811, Asp845, Arg849, and Asn850, to name a few [24]. In the docking procedure, we considered the distance d ≤ 4 Å between a proton and a heteroatom of the adjacent heterocyclic molecule as an important factor that allows the hydrogen bonds to be formed. Thus, fitting the first poses of the ligands resulted in the formation of several bonds between the analyzed derivatives and amino acids within the cavity of the kinase domain of HER2 protein. On this account, we observed contact of the carboxyl group within the OA with Arg849 (2.970 and 3.160 Å), Asp845 (3.110 Å), and relative proximity of the phenylig ring of Phe731 (3.236 Å). On the other hand, in the case of HIMOXOL, the following type of interaction between the ligand and particular amino acid were observed as follows: N-O(H)⋯H-N_Arg811_ (3.030 Å) and O⋯H-N_Arg756_ (3.703 Å), and the Br-HIMOLID, however, was able to form the following type of contacts: N-O(H)⋯H-N_Arg811_ (3.080 Å), and additionally Br⋯H-N_Arg849_ (2.796 Å), C=O⋯H-N_Phe731_ (3.030 Å), and Br⋯H-N_Asn850_ (3.539 Å). The proximity of the phenyl ring of Phe731 (3.344 Å) was noticed in this case as well. The resulted data proved the Br-HIMOLID hetarene—in comparison with other ones—was able to form stronger contacts and therefore to be more potent to interact with the protein.

Based on the data from the docking protocol, the additional SAPT (symmetry-adapted perturbation theory) analysis (Psi4 software [25]) of ligand-amino acid complexes was performed, and the interaction energy was estimated. It is noteworthy that in this step we focused only on ligand–amino acid complexes with neutral charges (closed-shell systems). Apart from ligand–amino acid total interaction energy calculations, on this account, we considered the following energetic components: the electrostatics, exchange, induction, and dispersion terms (Tables S1–S3 in the Supplementary Materials). As a result, it turned out that regarding the possible interaction with Asp845 the lowest (more negative) values of the total energy SAPT0 (−2.521 kcal mol^−1^) was obtained in the case of HIMOXOL, despite this ligand–amino acid distance exceeded 4 Å, and was caused basically by the dispersion term (−3.353 kcal mol^−1^). Even greater contribution of the dispersion term to the interaction with Asp845 was noticed in presence of Br-HIMOLID (−5.630 kcal mol^−1^), and the total value of the interaction energy (3.233 kcal mol^−1^) was significantly lower in comparison with the OA acid (42.448 kcal mol^−1^). The important contribution of electrostatic and dispersion terms to the ligand–amino acid interaction was observed for all ligands and Phe731; however, the values of the total interaction energy pointed the HIMOXOL and Br-HIMOLID heterocycles to be more potent. Moreover, considering the interaction of analyzed derivatives within the 3pp0.pdb protein our results proved that Br-HIMOLID seemed to be most potent to interact with Asn850 (total interaction energy was −0.316 kcal mol^−1^), which was in good agreement with the results of the docking approach, and was significantly caused by the contribution of the dispersion energetic term (−0.888 kcal mol^−1^).

**Figure 2:**
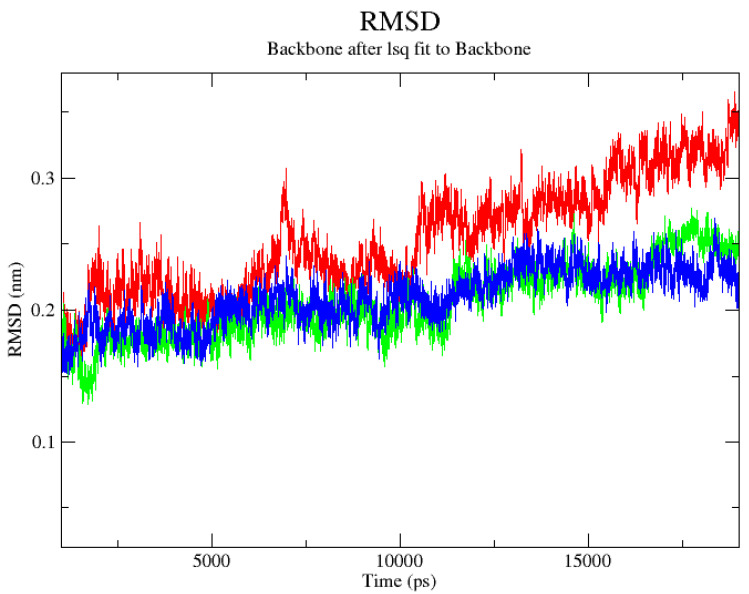
The RMSD plot for the ligand within ligand–protein complex during the MD productive phase was calculated for the complex of OA (red), HIMOXOL (green), or Br-HIMOLID (blue) and the 3pp0.pdb cavity.

Next, the 20-ns-long molecular dynamics (MD) simulations were performed to explore the stability of binding modes of ligands in the kinase domain of HER2 protein. The time-evolution of RMSD values of the ligand in the ligand–protein complexes is shown in Figure 2.

The ligand RMSD plot shows that the docking poses of all ligands, apart from OA acid, are stable inside the pocket. The maximum trajectory deviation was running from 2.480 (HIMOXOL) to 2.510 Å (Br-HIMOLID), and the average value of the RMSD factor was ca. 2 Å. These two observed RMSD fluctuations were practically analogous, whereas for OA, this parameter changed in a 1.950–3.53 Å range. Due to a large number of possible contacts, we initially chose to represent the results of the MD simulations in the form of radial distribution functions (RDFs) relative to particular ligand–amino acid contacts as supporting tools for hydrogen bonding analysis (Figures S1–S5 in the Supplementary Materials). We observed that for Arg811, Asp845, and Asn850 the maximum of the RDF curve involved HIMOXOL (15.512, 10.065, 7.015 and 10.065 Å for Arg811, Asp845, Arg849, and Asn850, respectively) and Br-HIMOLID derivatives (14.423, 8.976, 6.144, and 9.847 Å for Arg811, Asp845, and Asn850, respectively), thus, more favorable than oleanoic acid, whereas the RDF curve for the interactions of Br-HIMOLID with Phe731 was of low intensity. Considering the half-lifetime of hydrogen bonds formed between analyzed compounds and 3pp0.pdb protein in the function of time (Figures S6–S8 in the Supplementary Materials), we noticed that for the Phe731 this parameter exceeded the value of 10 ns of MD production in case of all heterocyclic ligands, but was not detected for OA and HIMOXOL regarding their interaction with Arg811. In the interaction of Br-HIMOLID with Arg849, this descriptor reached only ca. 1 ns in our simulation. We can draw these conclusions by supporting analysis of donor-acceptor distances within HBs (Figures S9–S11 in the Supplementary Materials), and for the Br-HIMOLID–Arg849 interaction (within the complex) we detected two values of distances: 2.969 and 3.406 Å, which proved that Br-HIMOLID was able to form with Arg849 electrostatic contacts with very short half-lifetime. As the distance between analyzed hetarenes and Asn850 exceeded the cut-off (3.500 Å) of the used software, the HBs related with this amino acid during trajectory was not detected.

Our results allowed us to assume that Arg811, Arg849, Asp845, and Asn850 seemed to be important for the interaction of Br-HIMOLID or HIMOXOL with the analyzed protein. The oleanane derivative with the bromine functionality was pointed on this account to be more potent in the interaction within the complex during MD simulation.

### 2.2. Effects of OA and Its Derivatives on Breast Cancer Cells Viability—MTT Assay

**Figure 3:**
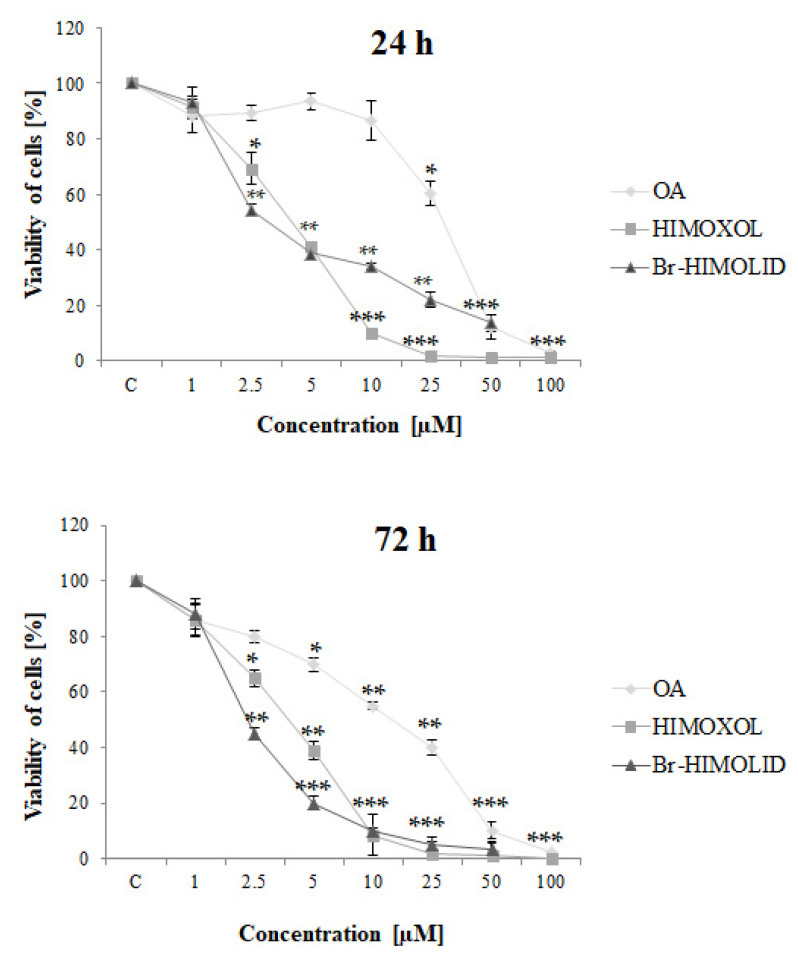
Contribution of oleanolic acid and its derivatives to breast cancer cells viability. SK-BR-3 cells were treated for 24 or 72 h with a high range of concentrations of studied compounds, i.e., 1–100 µM. The mean of three experiments ±SD is shown. Statistically significant difference versus control samples is shown: * *p* < 0.05; ** *p* < 0.005; *** *p* < 0.001.

MTT assay was performed to assess the cytotoxic effect of oleanolic acid and its derivatives on SK-BR-3 cells (Figure 3).

The IC_50_ values (Table 1) revealed that both studied derivatives showed higher cytotoxicity than maternal compound, OA, both after 24 h and 72 h of treatment.

HIMOXOL and Br-HIMOLID decrease the viability of studied cells much more efficiently than the maternal compound, i.e., the 1× IC_50_ values for 24 h incubation time were around six times lower for the derivatives than for the OA. Similarly, 72 h incubation revealed three or four times higher toxicity of the HIMOXOL and Br-HIMOLID than OA, respectively. The effect was dose- and time-dependent, and it was shown that HIMOXOL was more cytotoxic in 24 h-treatment while Br-HIMOLID was more toxic in 72 h-treatment.

Based on 24 h MTT results we chose the concentrations of the compounds to be tested in further experiments. We selected the range of three concentrations to assess the metabolic effect of 0.5× IC_50_ (slightly cytotoxic; the first value that showed significant toxicity), 1× IC_50_, and 1.5× IC_50_, respectively (Table 2).

### 2.3. Influence of OA and Its Derivatives on the Colony Formation of SK-BR-3 Cells

**Figure 4:**
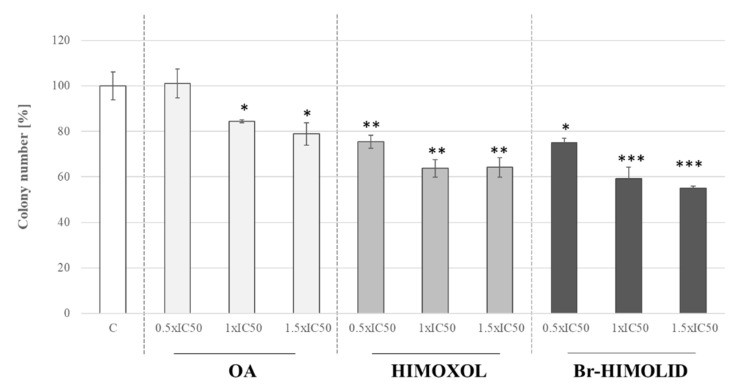
Influence of OA and its derivatives on the colonies formation of SK-BR-3 cells. The cells were plated in each well of a 6-well plate at 100 cells/mL and were allowed to adhere for 24 h. They were then treated with studied compounds in three different concentrations, corresponding to 0.5× IC_50_, 1× IC_50_, or 1.5× IC_50_ for 24 h. After the specified time interval, media was replaced and the cells were grown for ten days with one media change on the fourth day. The colonies formed were fixed with formaldehyde and stained with crystal violet. The wells were then washed, air-dried, and the colonies were enumerated. The experiment was repeated at least two times; × ±SD, *p* < 0.05. Statistically significant difference versus control samples is shown: * *p* < 0.05; ** *p* < 0.005; *** *p* < 0.001.

The clonogenic assay is an in vitro cell survival assay based on the ability of a single cell to form a colony [26]. It also reflects the genotoxic potential of compounds. In our study, OA, HIMOXOL, and Br-HIMOLID were applied to studied cells. In cells treated with the OA at 0.5× IC_50_, no significant alteration was reported, relative to control, untreated cells. When the cells were treated with OA at a concentration of 1× IC_50_ or 1.5× IC_50_ a significant decrease of colony-forming potential in SK-BR-3 cells was observed (around 20% in both cases, *p* < 0.05). Treatment of the cells with HIMOXOL or Br-HIMOLID provoked a significantly diminished clonogenic potential in all applied concentrations (0.5× IC_50_, 1× IC_50_, 1.5× IC_50_) up to around 40% in both cases (Figure 3; *p* < 0.05) in a dose-dependent manner (Figure 4).

### 2.4. The Effect of OA and OA Derivatives on the Basal Level of HER2 and Cell Cycle in SK-BR-3 Breast Cancer Cells

**Figure 5:**
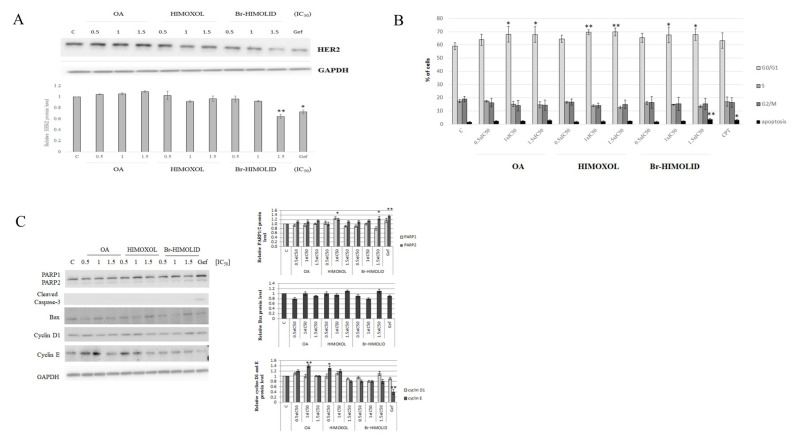
Evaluation of cell cycle modulation and proapoptotic potential of OA and its derivatives. SK-BR-3 cells were treated with studied compounds and 0.5×, 1×, or 1.5× IC_50_ values were applied followed by immunodetection. (**A**) Immunoidentification of target proteins was subjected to densitometry analysis. (**B**), normalized to GAPDH. Gefitinib (Gef, 20 µM) was used as a positive control. The mean value of three experiments ±SD is shown; *p* < 0.05. (**C**). Statistically significant difference versus control samples is shown: * *p* < 0.05; ** *p* < 0.005.

Since we decided to evaluate the influence of OA and OA-derivatives on HER2-positive cells that was supported by some literature premises, we performed immunoidentification of HER2 to verify any potential association between this receptor level and studied compounds. Treatment of the cells with OA or its derivatives (0.5× IC_50_, 1× IC_50_, or 1.5× IC_50_) revealed that only Br-HIMOLID significantly attenuated the accumulation of the target protein in studied cells (Figure 5A).

However, the effect was only noticeable at the highest applied concentration (1.5× IC_50_, i.e., 5.8 µM), suggesting some non-specific interaction that was probably reflecting the survival interference. For this reason, further experiments were performed to evaluate the contribution of the studied compounds to the cell cycle of SK-BR-3 cells.

To verify if the decreased viability of SK-BR-3 cells (MTT and clonogenic tests) is associated with cell cycle alteration, a cytofluorometric analysis was performed. The cells were treated with three different concentrations of OA, HIMOXOL, or Br-HIMOLID, corresponding to 0.5× IC_50_, 1× IC_50_, 1.5× IC_50_. The cell cycle analysis revealed an increased accumulation of the SK-BR-3 cells in G0/G1 phase (over 10%) after treatment with the higher compounds concentrations (1× IC_50_ and 1.5× IC_50_). It was accompanied by a significant decrease in the number of cells in the S phase. Moreover, Br-HIMOLID revealed a two-fold increase in the number of apoptotic cells (1.5× IC_50_) relative to control, untreated cells (3.7% vs. 1.7%). This effect was similar to the effect observed in cells treated with camptothecin (positive control, 20 µM, time 24 h) (Figure 5B).

### 2.5. Verification of Cell Cycle Modulation and Apoptosis Induction by Br-HIMOLID

In the MTT assay, we observed a significant decrease in cells viability, which was confirmed in the colony-forming assay. Moreover, cell cycle analysis revealed that HIMOXOL and Br-HIMOLID caused the arrest of the cells in the G0/G1 phase. Additionally, the highest Br-HIMOLID concentration (1.5× IC_50_) induced apoptosis (Figure 5B). Analysis of PARP, which is a substrate for caspase-3 revealed a significant, showed a slight decrease in 116 kDa subunit with an increase in 89 kDa subunit of PARP (both over 20%) only in cells treated with the highest concentration of Br-HIMOLID (1.5× IC_50_). Proapoptotic protein Bax also revealed an increase after treatment of cells only with the highest concentration of Br-HIMOLID (up to 15%). Assessment of proteins associated with the cell cycle, i.e., cyclin D1, and cyclin E, was also performed. Analysis of cyclin D1 revealed an increase in cells treated with the highest concentration of Br-HIMOLID (over 15%). However, the assessment of cyclin E showed an increase in cells treated with OA in the concentration corresponding to 1× IC_50_ (up to 40%) and HIMOXOL in the concentration corresponding to 0.5× IC_50_ and 1× IC_50_ (up to 30% and 20%, respectively) (Figure 5C).

### 2.6. Evaluation of Autophagosomes Formation and Verification of Autophagy Pathways

Since the cytotoxicity was not fully reflected by apoptosis proteins analysis, in the next step, we directed our investigation on the autophagy process.

**Figure 6:**
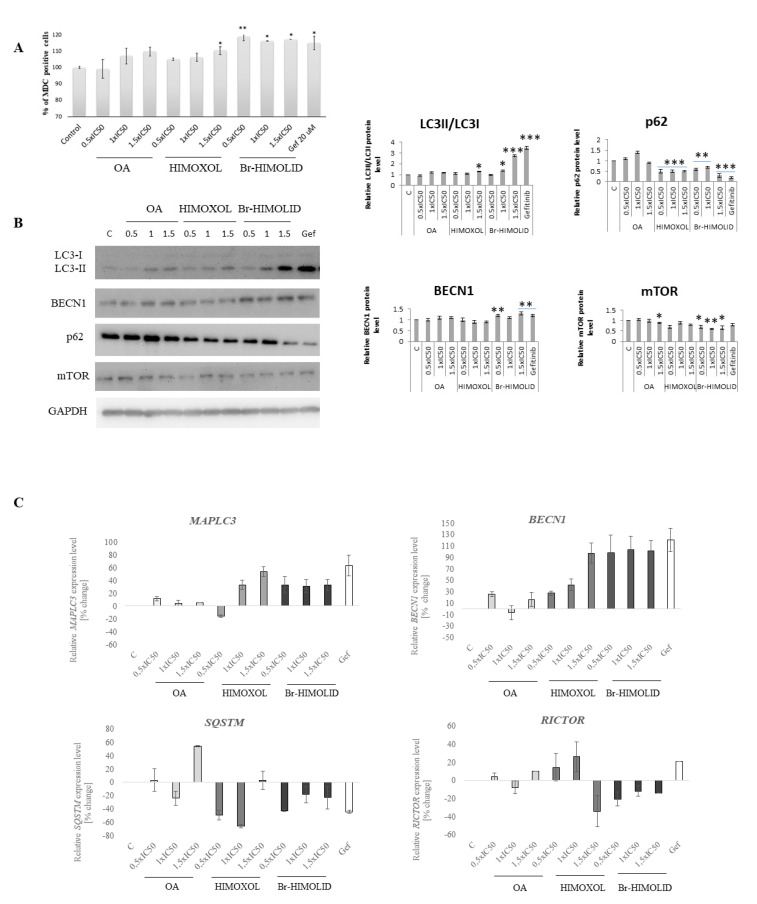
Evaluation of autophagy induction by OA and its derivatives. SK-BR-3 cells were treated with studied compounds, i.e., 0.5× 1× or 1.5× IC_50_ respective values were applied followed by monodansylcadaverine (MDC) staining (mean fluorescence, MFI, detection) and immunodetection. (**A**) Bars represent relative MFI of MDC staining in the indicated samples versus corresponding controls; B, immunoidentification of target proteins was subjected to densitometry analysis normalized to GAPDH. Gefitinib (Gef, 20 µM) was used as a positive control. The mean of three independent experiments is shown with *p* < 0.05 calculated from paired data. × ±SD, *p* < 0.05 (**B**). Statistically significant difference versus control samples is shown: * *p* < 0.05; ** *p* < 0.005; *** *p* < 0.001. (**C**) Evaluation of autophagy induction by OA and its derivatives in breast cancer cells. Cells were treated with studied compounds, i.e., 0.5×, 1×, or 1.5× IC_50_ respective values for 24 h. Gefitinib (Gef, 20 µM) was used as a positive control. Expression of target genes (MAPLC3, ECN1, SQSTM (p62), RICTOR (mTOR) at the mRNA level was performed using qPCR (C). GAPDH was used as a reference gene. The mean of three independent experiments ±SD is shown with *p* values calculated from paired data. Statistically significant difference versus control samples is shown: * *p* < 0.05; ** *p* < 0.005; *** *p* < 0.001.

To verify autophagy induction, staining with MDC, an autofluorescent molecule that accumulates in acidic autophagic vacuoles [27], was performed. This experiment was carried out using flow cytofluorometry and MFI assessment, relative to control cells. As a positive autophagy control, gefitinib (20 µM) was used. Cells treated with OA or HIMOXOL in the highest applied concentrations revealed an increase in the MFI’s value. Moreover, Br-HIMOLID in all applied concentrations (1.94–5.81 µM, corresponding to 0.5× IC_50_–1.5× IC_50_) revealed an increase of autophagy marker, up to 20% (Figure 6A). Although this method is not selective, it provides some basic evaluation of autophagy. Thus, we performed another experiment to verify autophagy induction in studied cells after treatment with OA and its derivatives.

### 2.7. Ability of Studied Semi-Synthetic Derivatives for Induction of Autophagy

Assay with monodansylcadaverine revealed induction of autophagy in SK-BR-3 cells treated with OA and its both derivatives. Thus, to confirm this observation, a Western blot analysis was performed. The specific autophagy marker consists of LC3 protein and its isoforms ratio (LC3II/LC3I); the LC3-II isoform is associated with the autophagosome membrane. Analysis of this protein revealed that Br-HIMOLID in the concentration corresponding to 1× IC_50_ and 1.5× IC_50_ increased LC3-II/LC3-I ratio (up to 270% relative to control). Moreover, assessment of the positive regulator of autophagy, beclin-1 showed an increase in the level of this protein (up to 30% relative to control). Simultaneously, analysis of the next two proteins, negatively involved in autophagy activation, p62 (SQSTM) and mTOR, revealed a decreased level of both proteins (by 30% and 70%, respectively) and confirmed induction of autophagy induction in SK-BR-3 cells (Figure 6B). These observations were also confirmed at the mRNA level in qPCR analysis of respective genes. In cells treated with HIMOXOL in a concentration corresponding to 1× IC_50_ and 1.5× IC_50_ and in all concentration range of Br-HIMOLID we observed overexpression of MAPLC3 (LC3) and BECN1 (beclin1). However, the mRNA analysis of SQSTM (p62) revealed downregulation of this gene in cells treated with HIMOXOL in the concentration corresponding to 0.5× IC_50_ and 1× IC_50_ (up to 60%) and a significant decrease of this gene expression in cells treated with 0.5× IC_50_ of Br-HIMOLID (over 40%). Moreover, downregulation of RICTOR (mTOR) gene has been observed in cells treated with the highest applied concentration of HIMOXOL (1.5× IC_50_, 35%) and the lowest of Br-HIMOLID (0.5× IC_50_, 20%) (Figure 6C).

### 2.8. OA and Its Derivatives Influence Migration of SK-BR-3—Wound Healing Assay

**Figure 7:**
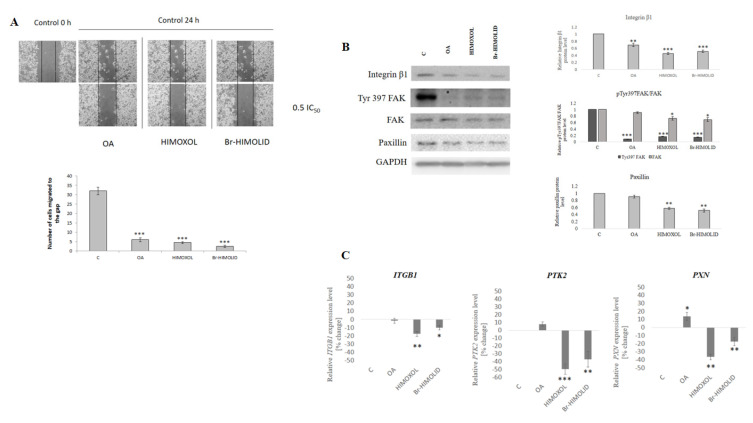
Influence of OA and its derivatives on migration of SK-BR-3 cells. (**A**) Effect of OA and its derivatives, HIMOXOL and Br-HIMOLID, on wound healing in SK-BR-3 breast cancer cells. Cells were scraped with a pipette tip and treated with OA or its derivatives in a concentration corresponding to 0.5× IC_50_ for 24 h. The photos represent cell migration under the microscope at 100 × magnification before (Control, 0 h) and after scratch. The experiments were repeated three times. *** *p* < 0.001 compared to the control group. (**B**) Contribution of OA and its derivatives to adhesion and migration of breast cancer cells. Evaluation of integrin β1, FAK, and paxillin protein accumulation in the SK-BR-3 cells (8 × 10^4^) was performed using immunodetection followed by densitometry analysis. Cells were treated with OA and its derivatives (Br-HIMOLID and HIMOXOL) in a concentration corresponding to 0.5× IC_50_ for 24 h. The mean of three independent experiments ±SD is shown with *p* values calculated from paired data. Statistically significant difference versus control samples is shown: * *p* < 0.05; ** *p* < 0.005; *** *p* < 0.001. (**C**) Contribution of OA and its derivatives to the expression of genes contributing to a migration-associated pathways in breast cancer cells. SK-BR-3 cells (8 × 10^4^) were treated with OA, Br-HIMOLID, or HIMOXOL for 24 h. Expression of target genes (integrin β1, FAK, and paxillin) was evaluated using specific primers and qPCR, relative to housekeeping gene GAPDH. Data from three independent experiments are shown as mean ±SD. The mean of three independent experiments ±SD is shown with *p*-values calculated from paired data. Statistically significant difference versus control samples is shown: * *p* < 0.05; ** *p* < 0.005; *** *p* < 0.001.

To verify if OA or its derivatives affect the adhesion and migration potential of SK-BR-3 cells, the wound healing assay was performed. After 24 h of treatment with a sub-cytotoxic concentration of all studied compounds (0.5× IC_50_), a scratch was performed using a pipette tip. Consequently, cells were treated with the studied compounds, and the number of cells grown in the area of the scratch was calculated. Interestingly, oleanolic acid provoked a significant decrease in cell migration (70% reduction of the cell number in the scratch area relative to control cells, i.e., 6 vs. 32 cells). At the same time, HIMOXOL reduced the migration potential by 80% (i.e., 4 vs. 32 cells), while Br-HIMOLID decreased the migration of SK-BR-3 cells by 95% (i.e., 2 vs. 32 cells) (Figure 7A).

### 2.9. Verification of Pathway Engaged in Anti-Migratory Potential

The anti-adhesive and anti-migratory potential of OA and its derivatives was verified by Western blot and qPCR analysis.

Immunoidentification of the proteins associated with focal adhesion signaling revealed that all the compounds applied in sub-cytotoxic concentration (corresponding to 0.5× IC_50_) significantly decreased integrin β1 level (up to 50% relative to control) and Tyr397 FAK (up to 80% relative to control). Additionally only the OA derivatives decreased total FAK (up to 30%) and paxillin (up to 50%, relative to control cells) proteins level (*p* < 0.05) (Figure 7B).

Assessment of the expression of genes contributing to migration-associated pathway revealed that treatment of studied cells for 24 h with HIMOXOL or Br-HIMOLID in applied sub-cytotoxic concentration, corresponding to 0.5× IC_50_ significantly decreased the expression of ITGB1 (up to 15–20%), PTK2 (up to 45%), and PXN (30% and 35%, respectively). When cells were treated with oleanolic acid, the expression status of ITGB1 and PTK2 was not significantly altered, however, the expression of paxillin (PXN) increased (by up to 30%).

## 3. Discussion

Breast cancer is one of the most common neoplasms among women, and the constantly growing morbidity and mortality confirm still insufficiently effective diagnostic and prognostic tools. The great difficulty in this area today is the lack of a sufficiently personalized therapy suitable for each type of cancer. The main goal of modern oncology is to predict the response of a specific type of breast cancer to the used drug. Compounds that specifically act on cancer cells without damaging normal cells are especially sought. Scientific reports indicate a broad spectrum of activity of substances belonging to pentacyclic triterpenes. One of the best-known representatives of this group is oleanolic acid, which has among others: anti-cancer properties [6]. However, this compound, despite its proven activities, has a poor water solubility which limits its applications [28]. Moreover, OA does not act selectively; therefore, there is a need to obtain or produce synthetic derivatives of oleanolic acid to increase its biological activity. Therefore, this study aimed was to investigate the mechanisms of action of OA and its two semi-synthetic derivatives: HIMOXOL and Br-HIMOLID in HER2-positive SK-BR-3 breast cancer cells. These compounds differ from the parental compound by their hydroxyimino, lactone, carboxylic acid moieties, and an additional bromine atom. These groups increase the effectiveness of the compound’s activity. It has been proven that the hydroxyimine moiety in the C3 position can both inhibit the proliferation of neoplastic cells and cause cell death—apoptosis, autophagy, or necrosis. It is worth mentioning an additional nitrogen atom, which increases the activity of the compounds. The published studies also indicate the importance of the imine group, showing antitumor, antibacterial, and antiviral properties [29]. On the other hand, the bromine atom that is part of the lactone derivative, shows antimicrobial properties, increases lipophilicity and biomedical application [30,31]. Moreover, the addition of other prosthetic groups to OA raises the possibility of reactions with other compounds, thereby increasing the biological activity of this triterpene [32].

The information about the anti-cancer properties of oleanolic acid derivatives is very sparse. The weak tumor-suppressive activity of OA was demonstrated in several cancer cell lines, including breast cancer MCF7 and MDA-MB-231 [33]. The results of our earlier studies indicate that the tested OA derivatives, HIMOXOL and Br-HIMOLID, showed higher efficiency of action than the oleanolic acid itself in other tumor cell lines of the mammary gland: MCF7, MDA-MB-231, MDA-MB-468, and T-47D. The above-mentioned cell lines represent different molecular subtypes of breast cancer, while in all these cells the effectiveness of HIMOXOL and Br-HIMOLID was reported to be higher than the parental compound (OA) [8,9,14]. In almost all previously studied breast cancer cell lines, HIMOXOL revealed higher cytotoxic, proapoptotic, and pro-autophagic effects than Br-HIMOLID, although the lactone derivative revealed slightly higher anti-migratory potential. Interestingly, their biological activity (survival inhibition) in non-cancerous cells of the breast gland, MCF-12A, was significantly lower [9]. However, the association between these derivatives and HER-2 receptor status has not been studied so far.

The SK-BR-3 cells represent a group of cells that reveal an aggressive phenotype. It is associated with the expression of HER2 that is a transmembrane receptor. It shows constitutive tyrosine kinase activity involved in increased cell growth, differentiation, survival, and migration [34,35].

### 3.1. OA Derivatives and HER2-Positive Cancer Cells Survival

In our studies, HIMOXOL and Br-HIMOLID revealed cytotoxicity in HER2 positive SK-BR-3 cells that was higher than the effect of OA. Moreover, the cytometric analysis showed cytostatic properties of both derivatives, and a slight pro-apoptotic activity of OA and Br-HIMOLID in studied cells. However, a high decrease of cells viability in applied dose-time conditions suggested induction of mechanisms other than apoptosis that contributed to the elimination of the SK-BR-3 cells. Multiple mechanisms and phenotypes compose programmed non-apoptotic cell death, including vacuole-presenting cell death (autophagy, entosis, and paraptosis), mitochondrial-dependent cell death (mitoptosis and parthanatos), iron-dependent cell death (ferroptosis), as well as other types, such as necroptosis [36]. However, due to the premises from our previous work and other reports [8,9,37], we concentrated on the assessment of autophagy process, which is intimately associated with eukaryotic cell death and apoptosis.

### 3.2. Balancing on the Edge

The amplification of HER2 genes is associated with the proliferation and progression of certain aggressive breast cells, which results from signal transduction mediated by the activation of mTOR and Ras/Raf/MEK/MAPK pathways, causing adverse biological characteristics and clinical outcomes [38]. One of the critical mechanisms that control cell metabolism provides cell homeostasis, and is regulated by the same mentioned above signaling pathways is autophagy. Dysfunctional autophagy is associated with many diseases. In cancer, this process can be neutral, tumor-suppressive, or tumor-promoting. It depends on surroundings conditions [39]. The available literature data suggest the autophagy is an important therapeutic target for cancer therapy. The currently used regimens of systemic treatment of patients with breast cancer involve the use of drugs, which actively involve the participation of autophagy signaling pathways [40]. Two groups of these drugs have distinguished the drugs that are responsible for this process inhibition and those which induce autophagy [41]. It is proven that both triterpenes as well as oleanolic acid and ursolic acid stimulate autophagy, by promoting mitochondrial metabolism alteration (mitophagy) in lung cancer cells [42]. In our studies, we observed an increase in autophagy after treatment of HER-2 positive breast cancer cells with HIMOXOL and Br-HIMOLID. The mechanism underlying is connected with mTOR and also LC3II/LC3I, p62, and beclin-1. The LC3-II protein isoform is the only well-characterized protein that is specifically localized to autophagic structures throughout the process from phagophore to lysosomal degradation [43]. Proteins engaged in the autophagy process are also p62—which consists of the cargo for autophagosome membrane and is downregulated after induction of autophagy; beclin 1 (BECN1)—a protein associated in autophagosome maturation; and mTOR—kinase that inhibits autophagy process [44]. The PI3K/Akt/mTOR pathway is an example of that, and it is involved in growth, proliferation, survival, motility, metabolism, and immune response regulation. Activation of this pathway is one of the main causes of cancer cell resistance to antitumor therapies [45]. In our studies we observed a significant decrease of mTOR kinase level after treatment of SK-BR-3 cells with HIMOXOL and Br-HIMOLID, which suggests that both studied derivatives (and especially Br-HIMOLID) revealed activity by this pathway, showing a proautophagic effect.

### 3.3. The Link between Adhesion and HER2 Pathway

The HER2 gene amplification in breast cancer is closely related to tumor-cell multiplication and invasion, resulting in focal progression and distant metastases as well as poor response to standard chemotherapy regimens [46]. Cell adhesion to ECM is central to the migration/invasion/metastasis process and involves largely integrins [47,48]. Integrin receptors, as mediators of cell–ECM interaction, not only provide physical links with the cytoskeleton but also transduce signals from the ECM to the cell, so crucial for several cellular processes including proliferation, survival, and migration [49].

One of the most important integrins signaling molecules recruited into focal adhesions upon cell–ECM contact is focal adhesion kinase (FAK), which affects multiple critical cellular processes such as cell survival, proliferation, and motility. FAK is phosphorylated and activated following integrin-mediated adhesion. The autophosphorylation on Tyr397 recruits and activates Src, which in turn further phosphorylates FAK in the activation loop, creating the fully active FAK enzyme [50]. The activated FAK enables the recruitment of other scaffold and signaling molecules, like paxillin to the focal adhesion sites, consequently activating the downstream cell signaling [51,52].

β1 integrin induces EGFR- or HER (ErbB2)-targeted therapy resistance. In sensitive cells, the inhibition of the ErbB receptor family by either antibodies or tyrosine kinase inhibitors (TKIs) blocks Erk and Akt pathway activation leading to cell death and cell growth inhibition. In resistant cells, β1 integrin or its associated extracellular matrix (ECM) proteins are often overexpressed, leading to the activation of β1-downstream pathways such as PI3K or FAK/Src. These pathways converge to activate the serine kinase Akt that promotes cell survival and cell growth [53].

It has been established that integrin- and HER2-signaling are functionally linked by sharing common signaling pathways that actively modulate cellular responses involved in cancer cell invasion [54]. It was found, for instance, that FAK signaling was increased by overexpressing HER2 in cells with low endogenous HER2 [55]. Similarly, overexpression of focal adhesion proteins involved in cell-substrate adhesion, such as integrins, increased HER2 signaling, and resistance to anti-HER2 therapy [56,57]. Vice versa, knockdown of integrins or inhibition of FAK signaling improved anti-HER2 therapy effectiveness [58,59].

Moreover, loss in cell adhesion will block the pro-survival integrin-dependent signaling pathways including PI3K/AKT, FAK, NFκB leading to a particular form of apoptosis named anoikis [60,61]. Resistance to anoikis favors tumor progression and promotes the development of metastasis [51,62].

In our experiments, we observed significant inhibition of SK-BR-3 cells migration after treatment with a sub-cytotoxic concentration of OA and its derivatives, which was also confirmed in the expression and proteins level status of integrin β1, FAK, and a scaffold protein, paxillin. This explains the inhibition of migration of cells and the anti-proliferative effect observed in MTT and clonogenic assays.

### 3.4. Summary and Perspectives

HER2-positive breast cancer is one of the most metastatic and invasive breast cancer, and it is associated with a poor prognosis. Based on an in silico analysis, we revealed that Br-HIMOLID or HIMOXOL derivatives seemed to be more effective in the interaction within the pocket of the HER2 protein in comparison with the oleanolic acid. These observations are following an in vitro study of the model of this type of cancer, i.e., SK-BR-3 cells, which showed that semisynthetic derivatives of OA, HIMOXOL, and Br-HIMOLID, could be used to efficiently target HER2-positive breast cancer cells. Both derivatives decreased the viability of studied cells, revealing cytostatic and proapoptotic effects. Importantly, the derivatives were more efficient than the maternal compound and Br-HIMOLID appeared to be more efficient than HIMOXOL. We also observed proautophagic potential of both OA derivatives and this effect was mediated by the mTOR/LC3/p62/BECN1 signaling pathway. Moreover, both OA derivatives significantly reduced migration of HER2-positive SK-BR-3 breast cancer cells and this effect was mediated by modulation of the integrin β1/FAK/paxillin pathway. Additionally, WB analysis revealed that treatment of SK-BR-3 cells with Br-HIMOLID resulted in a significant reduction of HER2 protein level. Referring to this work and to previously published results [9] that showed no significant effect of OA, HIMOXOL, and Br-HIMOLID on non-cancer MCF-12A breast cells, we might conclude that both oleanolic acid derivatives might constitute an important element in planning further HER2-positive-targeted breast cancer therapy strategy.

## 4. Materials and Methods

### 4.1. Compounds and Reagents

**Figure 8:**
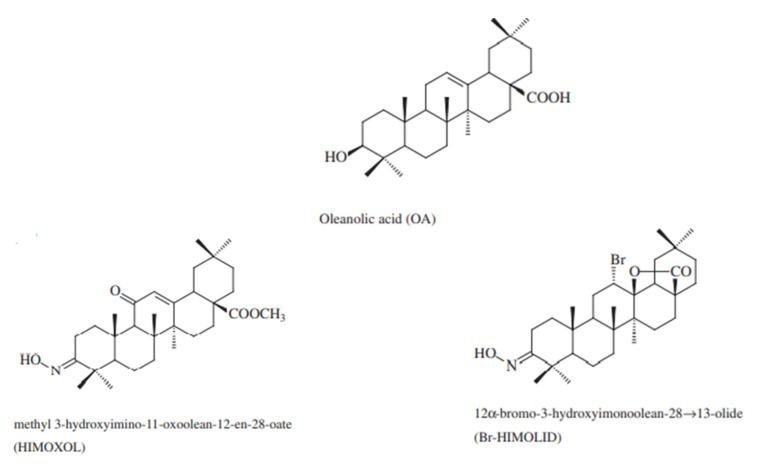
Structure of studied compounds.

Oleanolic acid was isolated from an industrial by-product obtained during the process of mistletoe herb essence production. Spectral data of the resulting chemicals were consistent with the data from the literature [63]. The semisynthetic OA derivatives, methyl 3-hydroxyimino-11-oxoolean-12-en-28-oate (HIMOXOL) and 12α-bromo-3-hydroxyimonoolean-28→13-olide (Br-HIMOLID) were synthesized as described in the earlier studies [64,65] at the Department of Organic Chemistry, Poznan University of Medical Sciences, Poland (Figure 8). Before use in the experiment, the derivatives were dissolved in DMSO and stored at 4 °C.

The cell culture medium, McCoy’s 5A, and fetal bovine serum were purchased from Lonza (Lonza, Verviers, Belgium). Rapamycin, DMSO, camptothecin, propidium iodide, RNAse, and MTT were obtained from Sigma-Aldrich (Sigma-Aldrich, Munich, Germany). Gefitinib was purchased from Cell Signaling Technology (Cell Signaling Technology, Danvers, MA, USA).

### 4.2. Computational Details

The optimization of all ligands using the Gaussian 16 C.01 program [20], QM approach, and density functional theory (DFT) formalism with the B3LYP/6-31G(d,p) approximation. The MD simulations (GROMACS 2016.4 software [66]) were carried out based on our previously published reports [67]. The crystal structure of the kinase domain of human HER2 (erbB2) (PDB entry: 3PP0) [18,19] with the resolution 2.250 Å was selected as the biological target as one of the most used for docking PDB version of the human HER2protein. To carry out docking simulation (using the AutoDock Vina package [21]), a grid box was defined to be of 10 Å size (centre _x = 16.491, centre_y = 17.032, centre_z = 25.759). The outputs (* *pdbqt* files) after the docking procedure (the projections of the 1st poses) were visualized using the LigPlot + v.2.2 software [22,23].

### 4.3. Cell Line and Cell Culture

The SK-BR-3 (ER-, PR-, HER2+) human breast cancer cell line was obtained from the American Type Culture Collection (ATCC, HTB-30). The SK-BR-3 cells were maintained in McCoy’s 5A medium supplemented with 10% fetal bovine serum. The cells were cultured in 5% CO_2_ at 37 °C and with saturated humidity. The cells culture medium was changed every 3–4 days.

### 4.4. Viability Assay

A total of 2.5 × 10^4^/mL growing cells were seeded into each well of the 96-well plates and compounds were added at 0.5–50 µmol/L. The solvent, DMSO at a concentration of 0.28%, was also applied as a control (Sigma-Aldrich, St Louis, MO, USA). Two duplicates were created for each concentration with a total volume of 100 µL per well. Then, 10 μL of MTT solution (5 mg/mL) was added to each well. The plates were incubated at 37 °C for 4 h followed by the addition of 100 μL solubilization buffer (10% SDS in 0.01 M HCl). Cell viability was quantified spectrophotometrically using a Labsystems Multiscan RC (Thermo, Champaign, IL, USA). Each experiment was repeated three times, IC_50_ values were calculated using the CampuSyn software (ComboSyn, Paramus, NJ, USA), and the standard deviation was calculated using the Microsoft Excel software (Microsoft, Redmond, WA, USA) [9,68].

### 4.5. Colony-Forming Assay

For the colony-forming assay, the cells were plated in each well of a 6-well plate at 100 cells/mL (200 per plate) and were allowed to adhere for 24 h. They were then treated with studied compounds in three different concentrations, corresponding to 0.5× IC_50_, 1× IC_50_, and 1.5× IC_50_ for 24 h. After the specified time point, the particles-containing media was replaced with fresh, complete media, and the cells were grown for ten days with one media change on the fourth day. The colonies formed were fixed with 4% formaldehyde (37 °C; 15 min) and stained with crystal violet (0.5% (*w*/*v*); 1 h, 25 °C). The wells were then washed with distilled water, air-dried, and the colonies were enumerated [68]. The experiment was repeated at least two times.

### 4.6. Cell Cycle Analysis by Flow Cytometry

SK-BR-3 cells were treated with indicated OA, Br-HIMOLID, and HIMOXOL concentrations for 24 h. The cells were collected using 0.25% trypsin (Sigma-Aldrich, St Louis, MO, USA), then washed and resuspended in 100 µL PBS containing 50 µg/mL propidium iodide and 25 µL of ribonuclease A (10 mg/mL) (Sigma-Aldrich, St Louis, MO, USA). After 1 h of incubation, flow cytometry analysis was performed (FACScan, Becton Dickinson, Franklin Lakes, NJ, USA). The percentages of the cell population in the subphases G0/G1, S, and G2/M were calculated from the histograms [9,69,70]. The experiment was repeated three times.

### 4.7. Detection of Autophagy by Flow Cytometry

Cells were sub-cultured in 6 cm-diameter Petri dishes at a quantity of 1 × 10^5^ cells per plate and incubated in appropriate media with OA, Br-HIMOLID, or HIMOXOL for 24 h. After treatment, the cells were washed twice with PBS, incubated with 0.05 mM monodansylcadaverine (MDC) (Sigma-Aldrich, St Louis, MO, USA) in PBS at 37 °C for 30 min and then washed three times with PBS at room temperature (RT). The mean fluorescence intensities (MFIs) from intracellular MDC were measured with flow cytometry (FACScan, Becton-Dickinson, Franklin Lakes, NJ, USA) [9]). The results are representative of two independent experiments ([MFIs] ± SD).

### 4.8. Wound Healing Assay

The anti-migratory effects of OA, Br-HIMOLID, and HIMOXOL, on human breast cancer cells (3 × 10^5^), were examined by wound healing assay. The cells were seeded in 6-well plates. After the cells reached confluency, an artificial scratch was made with a pipette tip in each well. The cells were washed with PBS followed by treatment with the noncytotoxic concentrations of the compounds (OA, Br-HIMOLID, or HIMOXOL) corresponding to the 0.5× IC_50_ values. The images of the experimental groups were taken at 24 h after treatment, and compared with control samples to quantify the migration ratio by cells enumeration. Each experiment was repeated two times [14].

### 4.9. Quantitative Gene Expression Assessment

The total RNA was extracted using a TRI reagent (Sigma-Aldrich, St. Louis, MO, USA) [71]. For the first-strand cDNA synthesis, 0.5 µg RNA was reverse transcribed using a Transcriptor First Strand cDNA Synthesis Kit (Roche Diagnostics, Indianapolis, IN, USA) as previously described [72]. Then, the cDNAs were subjected to quantitative real-time PCR analysis. The primers for LC3 (MAPLC3), p62 (SQSTM), Beclin-1 (BECN1), mTOR (RICTOR), integrin β1 (ITGB1), FAK (PTK2), and paxillin (PXN) were obtained from the commercially available primers’ set: Human Autophagy Primer Library and Human Adhesion Primer Library (HATPL-1 and HCAD-I, RealTimePrimers, Elkins Park, PA, USA). The primers used for GAPDH in this study were GAPDH 5′-TTCGTCATGGGTGTGAACC-3′ (forward) and 5′-GATGATGTTCTGGAGAGCCC-3′ (reverse). Real-time PCR was performed using SYBR Green PCR Master Mix (Roche Diagnostics, Indianapolis, IN, USA) in a reaction containing 0.25 µM of each primer, 0.5 µL template cDNA, and 1 × SYBR Green. The PCR was run at 94 °C for 10 min followed by 50 cycles at 94 °C for 20 s, 59 °C for 20 s, and 72 °C for 20 s. GAPDH was used as an internal control as previously described [69]. Gene expression was calculated using the comparative threshold cycle (2 △△CT) method. The experiments were performed in triplicate and the results show a mean value ± SD.

### 4.10. Immunodetection

The SK-BR-3 cells were treated for 24 h with three concentrations of Br-HIMOLID or HIMOXOL, corresponding to 0.5× IC_50_, IC_50_, and 1.5× IC_50_ (Table 2). Whole-cell extracts were prepared using a modified RIPA lysis buffer (50 mM Tris–HCl, pH 8.0, 150 mM NaCl, 1% NP40, 0.1% SDS, 100 mM PMSF, 25 μg/mL Na3VO4, 25 μg/mL NaF, 25 μg/mL leupeptin, and 25 μg/mL aprotinin). The protein concentration was measured using a Bradford assay (Sigma-Aldrich, St. Louis, MO, USA), and 40 μg of each extract was loaded onto SDS-PAGE gels. Western blotting was performed according to the standard procedure using a PVDF membrane (Pierce Biotechnology, Rockford, IL, USA) [70]. The following antibodies were used for detection: anti-MAPLC3, anti-mTOR, anti-BECN1, anti-p62, anti-Bax, anti-PARP1/2, anti-cleaved caspase-3, anti-integrin β1, anti-paxillin, anti-pFAK Tyr-397, anti-FAK (Cell Signaling Technology, Danvers, MA, USA), and anti-cyclin D1, anti-cyclin E and anti-GAPDH (Santa Cruz Biotechnology, CA, USA); 1 μg/mL of each primary antibody was used in the blotting solution. The proteins were visualized using the SuperSignal^®^ West Pico Chemiluminescent Substrate (Pierce Biotechnology, Rockford, IL, USA). The optical density (Arbitrary Units) of the bands was measured using the VisionWorks software (NVIDIA, Santa Clara, CA, USA). Representatives of at least two experiments are shown in Figure 5A,C, Figure 6B, and Figure 7B.

### 4.11. Statistical Analysis

The data are means from at least three separate experiments unless otherwise specified. Statistical analysis was performed by one-way ANOVA (GraphPad Prism 5, San Diego, CA, USA). A *p*-value < 0.05 was considered to be indicative of a significant difference.

## Figures and Tables

**Table 1:** Comparison of OA and its two semisynthetic derivatives cytotoxicity in SK-BR-3 cells. The IC_50_ values were calculated based on the dose-response curves assessed by the MTT assay for 24 and 72 h.

Compound Time	IC_50_ [µM]		
	OA	HIMOXOL	Br-HIMOLID
24 h	22.5 ± 1.27	3.89 ± 0.28	3.87 ± 0.35
72 h	11.31 ± 3.26	3.67 ± 0.25	2.56 ± 0.36

**Table 2:** The concentration range of compounds applied in all experiments.

IC_50_	[µM]/24 h		
	OA	HIMOXOL	Br-HIMOLID
0.5× IC_50_	11.25	1.95	1.94
1× IC_50_	22.5	3.89	3.87
1.5× IC_50_	33.75	5.84	5.81

## Data Availability

The data is contained within this article and Supplementary Materials.

## Supplementary Materials

The following are available online at https://www.mdpi.com/article/10.3390/ijms222011273/s1.
