# Supplementary material for: Oleanolic Acid’s Semisynthetic Derivatives HIMOXOL and Br-HIMOLID Show Proautophagic Potential and Inhibit Migration of HER2-Positive Breast Cancer Cells In Vitro
Source: Int J Mol Sci. 2021 Oct 19;22(20):11273. doi: 10.3390/ijms222011273 (PMC8538366; doi:10.3390/ijms222011273)
Supplement: Supplementary file 1 [file ijms-22-11273-s001.zip › ijms-1405418-supplementary-for XML conversion.pdf]

International Journal of
Molecular Sciences

MDPI

Oleanolic Acid's Semisynthetic Derivatives HIMOXOL and Br-HIMOLID Show Proautophagic Potential and Inhibit Migration of HER2-Positive Breast Cancer Cells In Vitro

Natalia Lisiak[1]*, Izabela Lewicka[2], Mariusz Kaczmarek[3,4], Jacek Kujawski[5], Barbara Bednarczyk-Cwynar[5], Lucjusz Zaprutko[5], Błażej Rubiś[1]

Supplementary Materials

Expanded analysis of the *in silico* studies on ligands interactions with the 3pp0.pdb protein.

Table S1. Calculated values of energetic terms [kcal/mol] regarding the ligand-amino acid interaction using the SAPT0 method for docked OA.

Amino acid	Energy				
	Electrostatics	Exchange	Induction	Dispersion	Total
Val734	0.035	0.000	-0.001	-0.066	-0.033
Ala751	-0.002	0.000	0.000	-0.005	-0.008
Leu726	0.124	0.000	-0.006	-0.071	0.048
Leu785	0.008	0.000	0.000	-0.003	0.005
Thr798	0.004	0.000	0.000	-0.004	0.000
Leu800	0.005	0.000	0.000	-0.004	0.001
Met801	0.019	0.000	0.000	-0.005	0.014
Cys805	0.058	0.000	-0.003	-0.092	-0.037
Ala847	-0.063	0.000	-0.003	-0.139	-0.205
Ala848	-0.064	0.000	-0.004	-0.134	-0.201
Asn850	-0.394	0.009	-0.058	-0.527	-0.969
Val851	0.027	0.000	-0.001	-0.039	-0.014
Leu846	-0.003	0.000	-0.004	-0.116	-0.123
Leu852	-0.003	0.000	0.000	-0.023	-0.026
Ile861	-0.007	0.000	0.000	-0.015	-0.022
Thr862	-0.048	0.000	-0.002	-0.037	-0.087
Thr911	0.042	0.000	-0.001	-0.017	0.024
Asp845	-47.395	127.998	-21.430	-16.725	42.448
Phe731	-50.325	162.091	-17.598	-31.849	62.317

Table S2. Calculated values of energetic terms [kcal/mol] regarding the ligand-amino acid interaction using the SAPT0 method for docked HIMOXOL.

Amino acid	Energy				
	Electrostatics	Exchange	Induction	Dispersion	Total
Val734	0.106	0.000	-0.009	-0.128	-0.031
Ala751	-0.083	0.000	-0.001	-0.009	-0.093
Leu726	0.564	0.000	-0.035	-0.138	0.391
Leu785	-0.027	0.000	0.000	-0.004	-0.032
Thr798	0.044	0.000	0.000	-0.006	0.038
Leu800	-0.069	0.000	-0.001	-0.007	-0.077
Met801	0.139	0.000	-0.002	-0.009	0.129
Cys805	0.361	0.001	-0.045	-0.273	0.044
Ala847	0.179	0.000	-0.013	-0.149	0.017
Ala848	0.186	0.000	-0.015	0.242	-0.071
Asn850	1.429	0.007	-0.110	-0.689	0.637
Val851	0.325	0.000	-0.010	-0.066	0.249
Leu846	-0.177	0.000	-0.007	-0.117	-0.301
Leu852	-0.150	0.000	-0.004	-0.044	-0.198
Ile861	-0.051	0.000	-0.002	-0.022	-0.075
Thr862	-0.168	0.000	-0.009	-0.058	-0.235
Thr911	0.189	0.000	-0.004	-0.022	0.164
Asp845	-0.869	2.092	-0.392	-3.353	-2.521
Phe731	-9.674	36.732	-4.356	-16.919	5.782

Table S3. Calculated values of energetic terms [kcal/mol] regarding the ligand-amino acid interaction using the SAPT0 method for docked Br-HIMOLID.

Amino acid	Energy				
	Electrostatics	Exchange	Induction	Dispersion	Total
Val734	-0.138	0.000	-0.009	-0.088	-0.234
Ala751	-0.037	0.000	-0.001	-0.006	-0.044
Leu726	-0.179	0.000	-0.007	-0.074	-0.260
Leu785	-0.030	0.000	-0.001	-0.003	-0.034
Thr798	0.009	0.000	0.000	-0.004	0.004
Leu800	-0.036	0.000	0.000	-0.005	-0.041
Met801	-0.002	0.000	-0.001	-0.006	-0.010
Cys805	-0.070	0.000	-0.005	-0.151	-0.227
Ala847	0.150	0.000	-0.006	-0.150	-0.006
Ala848	0.069	0.000	-0.005	-0.238	-0.173
Asn850	0.549	0.183	-0.161	-0.888	-0.316
Val851	0.010	0.000	-0.003	-0.055	-0.049
Leu846	-0.164	0.000	-0.002	-0.106	-0.272
Leu852	-0.040	0.000	-0.002	-0.032	-0.074
Ile861	-0.006	0.000	-0.001	-0.019	-0.025
Thr862	0.029	0.000	-0.006	-0.051	-0.027
Thr911	0.033	0.000	-0.002	-0.021	0.010
Asp845	-7.164	18.021	-1.994	-5.630	3.233
Phe731	-12.962	43.580	-6.083	-13.375	11.159

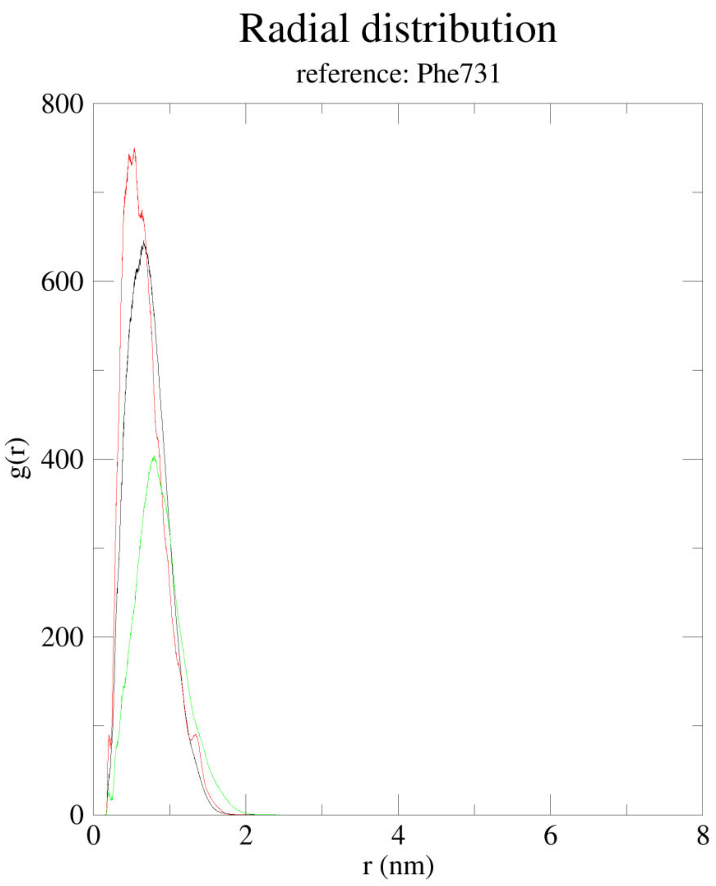

Figure S1. Radial distribution functions (RDFs) for OA (black), HIMOXOL (red) or Br-HIMOLID (light green) and Phe731 obtained from the MD simulation.

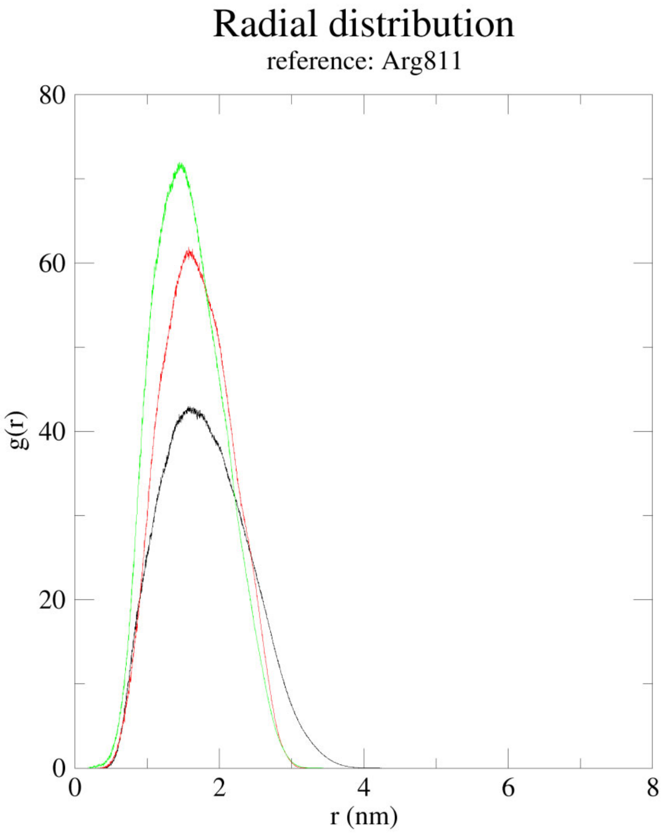

Figure S2. Radial distribution functions (RDFs) for OA (black), HIMOXOL (red) or Br-HIMOLID (light green) and Arg811 obtained from the MD simulation.

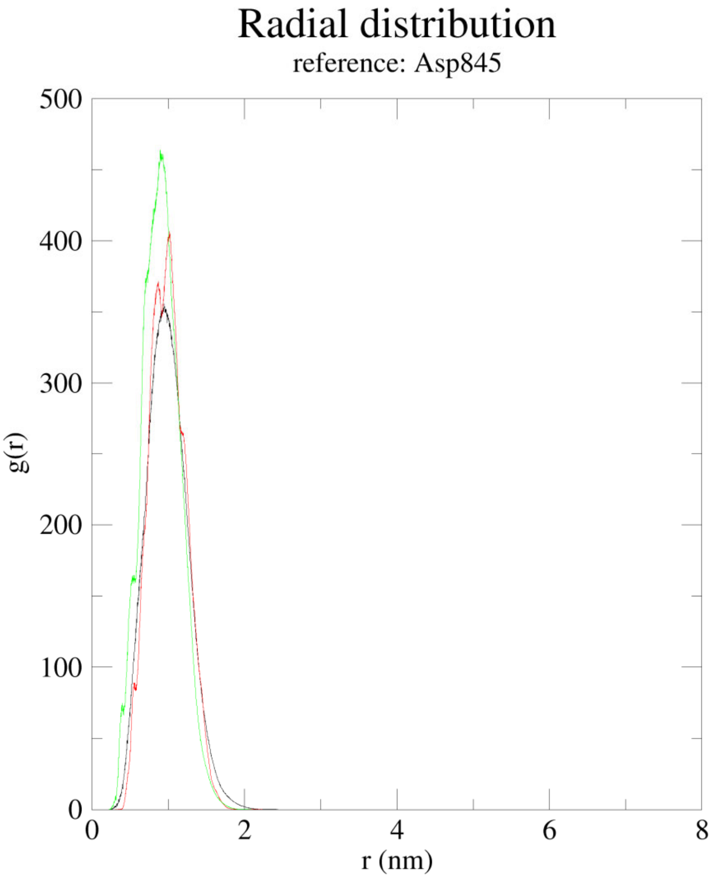

Figure S3. Radial distribution functions (RDFs) for OA (black), HIMOXOL (red) or Br-HIMOLID (light green) and Asp845 obtained from the MD simulation.

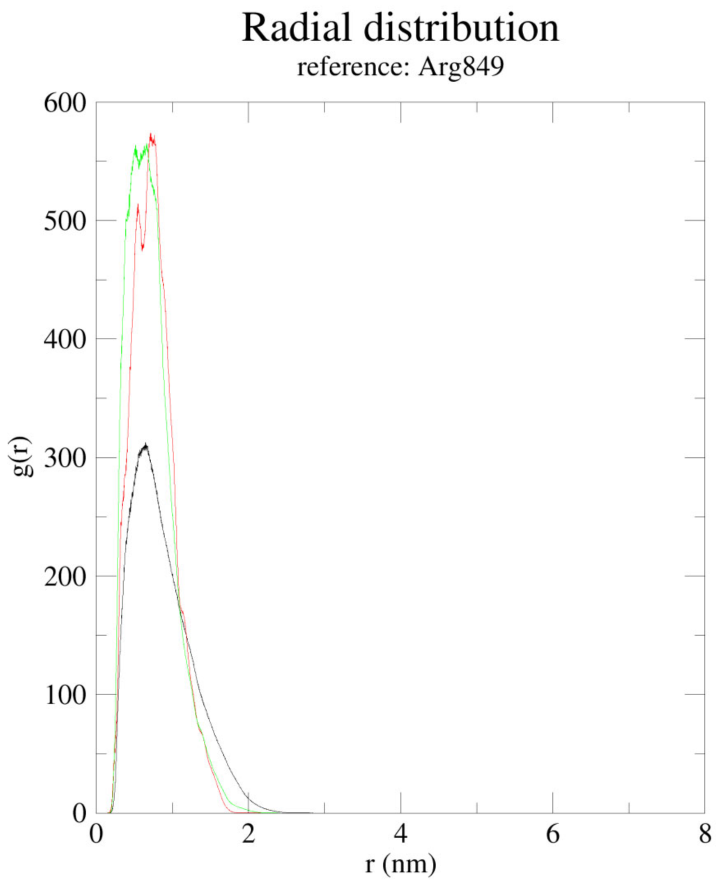

Figure S4. Radial distribution functions (RDFs) for OA (black), HIMOXOL (red) or Br-HIMOLID (light green) and Arg849 obtained from the MD simulation.

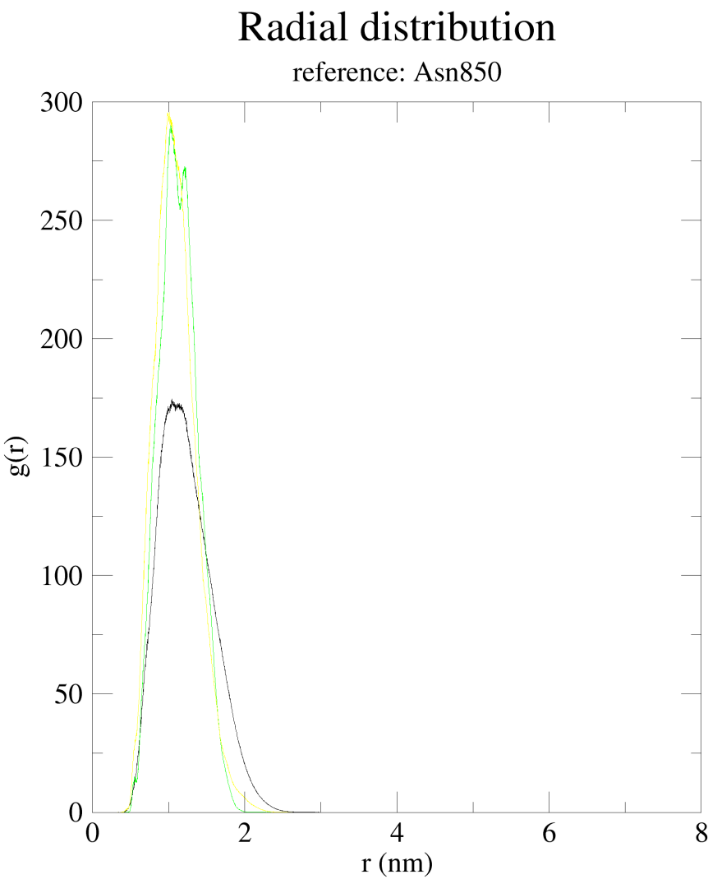

Figure S5. Radial distribution functions (RDFs) for OA (black), HIMOXOL (red) or Br-HIMOLID (light green) and Asn850 obtained from the MD simulation.

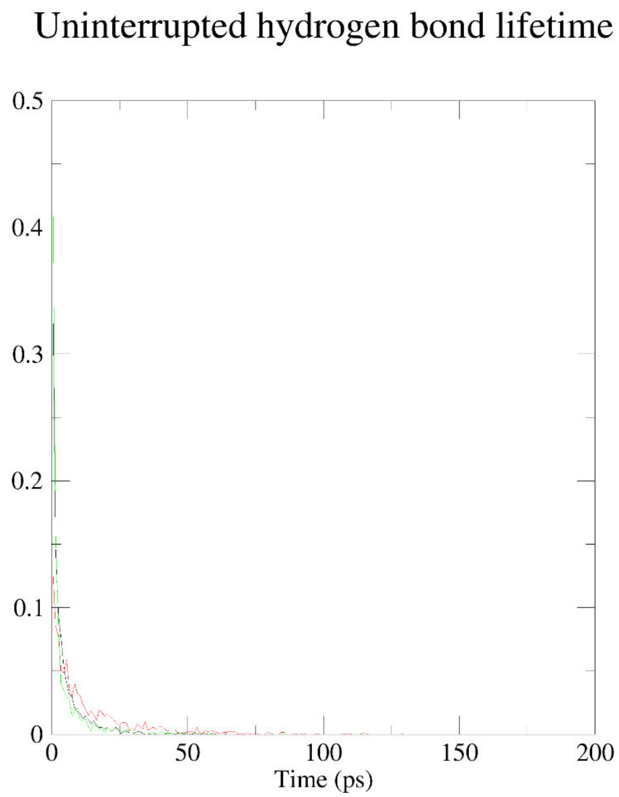

Figure S5. HBs lifetime for OA (black), HIMOXOL (red) or Br-HIMOLID (light green) and Asn850 obtained from the MD simulation.

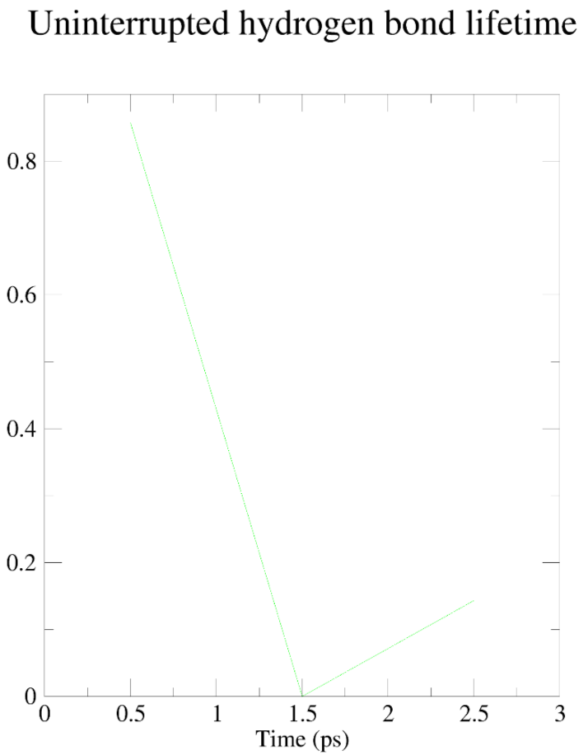

Figure S7. HBs lifetime for OA (black), HIMOXOL (red) or Br-HIMOLID (light green) and Arg811 obtained from the MD simulation.

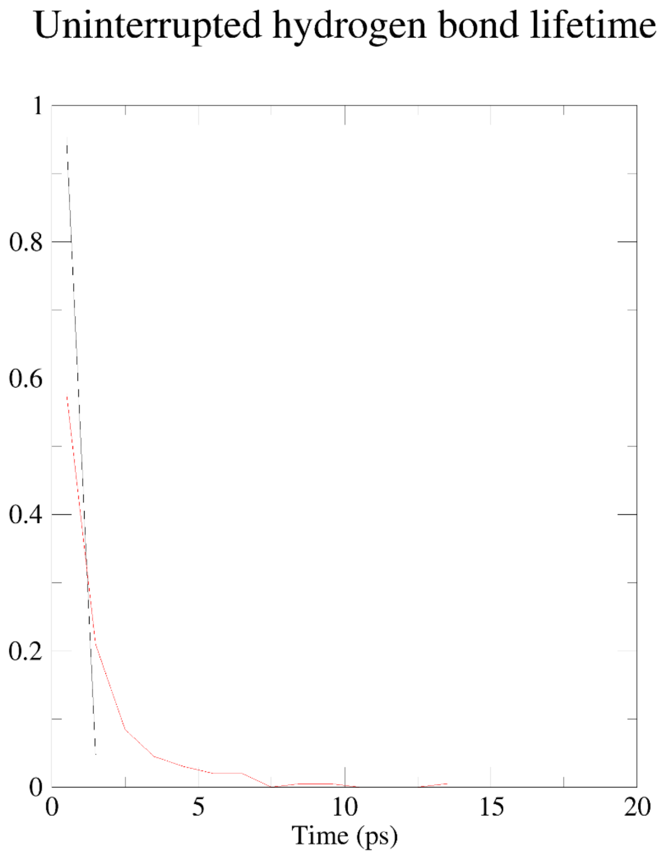

Figure S8. HBs lifetime for OA (black), HIMOXOL (red) or Br-HIMOLID (light green) and Arg849 obtained from the MD simulation.

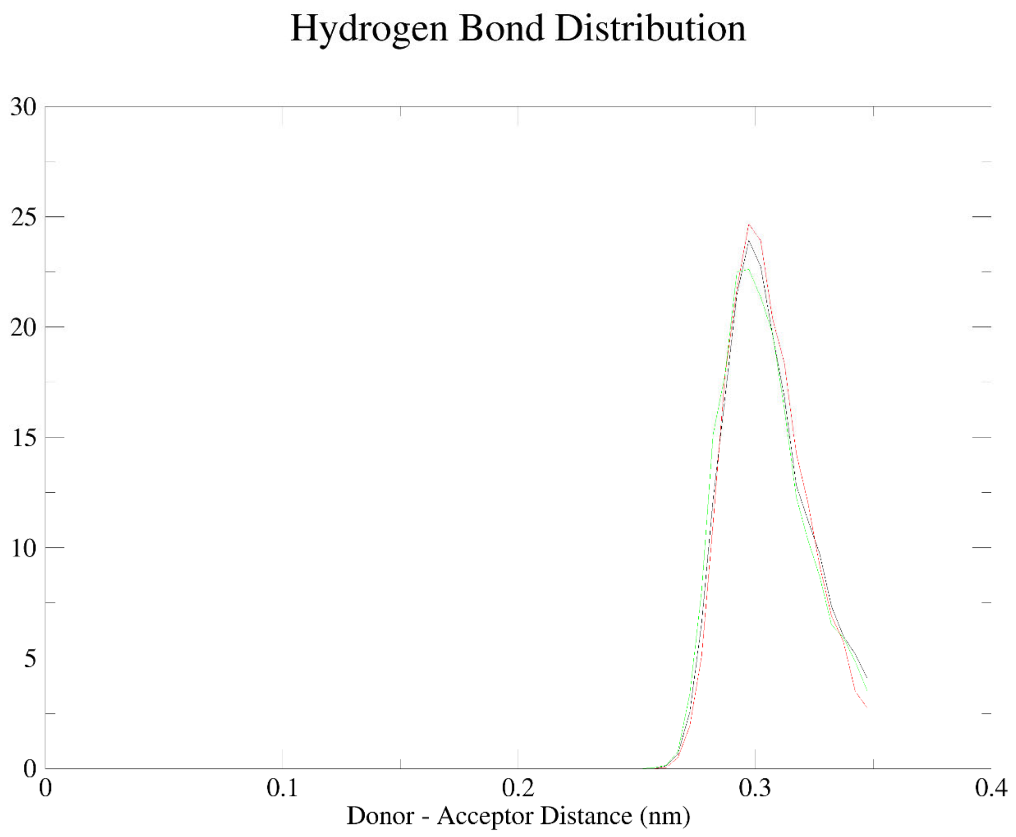

Figure S9. Donor–acceptor distances within HBs for OA (black), HIMOXOL (red) or Br-HIMOLID (light green) and Phe731 obtained from the MD simulation.

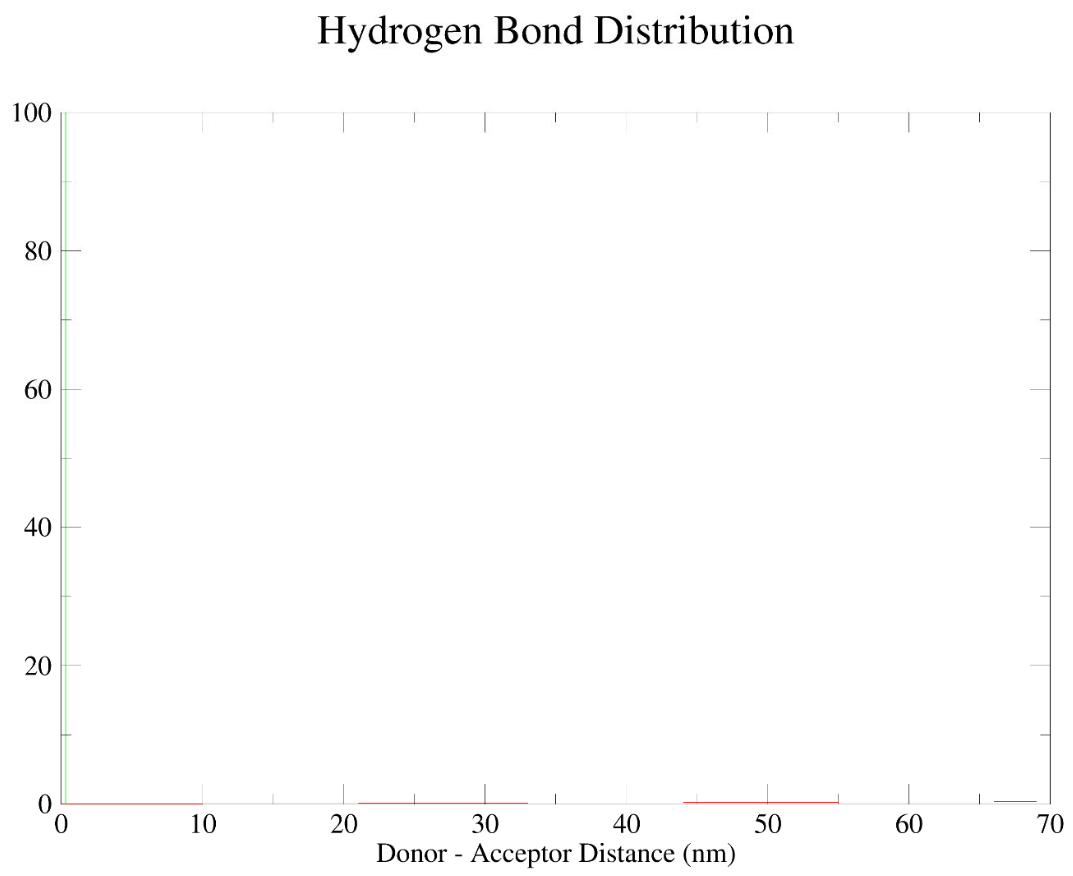

Figure S10. Donor–acceptor distances within HBs for OA (black), HIMOXOL (red) or Br-HIMOLID (light green) and Arg811 obtained from the MD simulation.

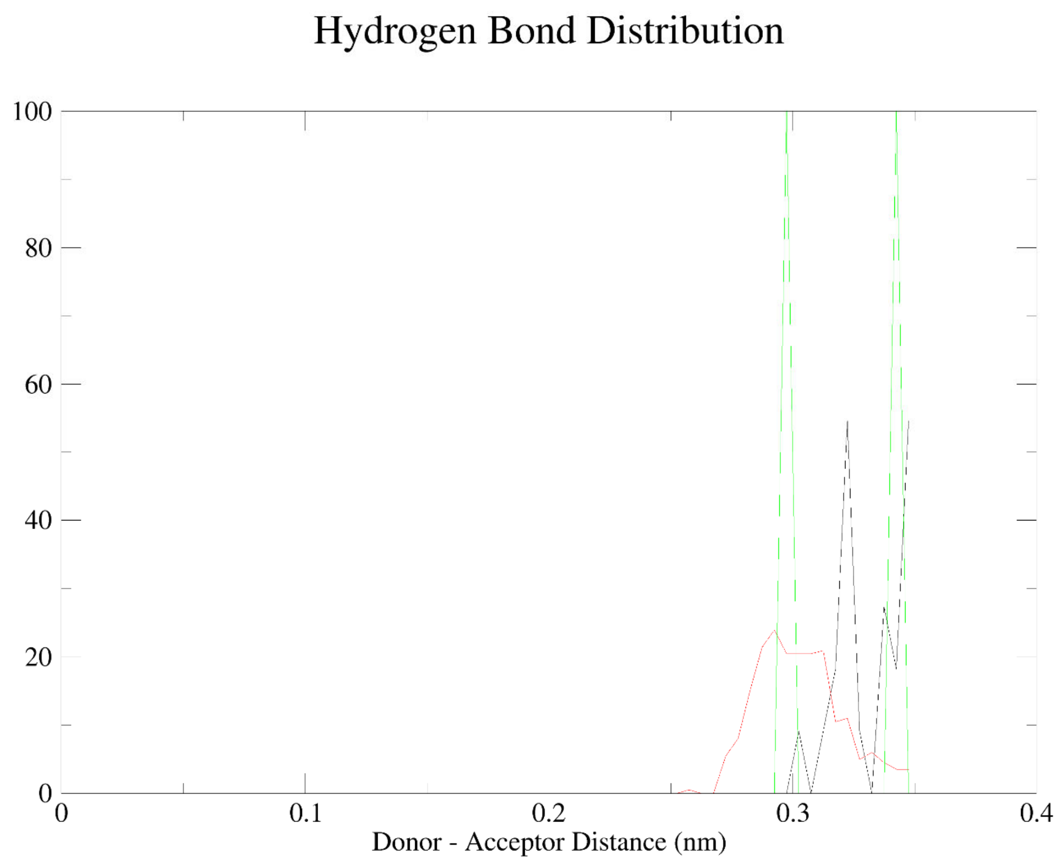

Figure S11. Donor–acceptor distances within HBs for OA (black), HIMOXOL (red) or Br-HIMOLID (light green) and Arg849 obtained from the MD simulation.
